# Genome sequences of *Methylobacterium* and *Methylorubrum* isolates from Cd-contaminated soils in the Tar Creek Superfund site

**DOI:** 10.1128/mra.00834-24

**Published:** 2025-01-21

**Authors:** Evan S. Grimes, Samuel J. Bergerson, Emily P. McHugh, David Monismith, James H. Campbell, Alisha G. Campbell

**Affiliations:** 1 Department of Natural Sciences, Northwest Missouri State University, Maryville, Missouri, USA; 2 Independent Researcher, Oklahoma City, Oklahoma, USA; 3 Loess Hills Research Center, Department of Natural Sciences, Northwest Missouri State University, Maryville, Missouri, USA; California State University San Marcos, San Marcos, California, USA

**Keywords:** *Methylobacterium*, *Methylorubrum*, cadmium resistance, Tar Creek Superfund site, Tri State Mining District

## Abstract

Soil bacteria were isolated from the Tar Creek Superfund site in the presence of cadmium. Eight of these strains belong to the genus *Methylobacterium*, whereas one strain belongs to the genus *Methylorubrum*. Here, we report drafts of their genome sequences and highlight cadmium-resistance genes required in an exceptionally contaminated environment.

## ANNOUNCEMENT

Mining activity (1) within the Tar Creek Superfund site left soils contaminated with Pb, Zn, and Cd (2) and altered microbial communities (3). The genomes presented here represent isolates from topsoil samples within this site and provide insight into survival mechanisms in heavy-metal-contaminated soils, particularly those with high Cd levels. Cd resistance has been documented in *Methylobacterium* species (4, 5), but the mechanism is unclear.

Isolates were enriched from samples taken in August 2015 within an 8.05 km radius of Picher, Oklahoma (2). One gram of each soil sample was diluted in 9 mL water, and 1 mL of this dilution was used to inoculate 0.5 × Nutrient Broth amended with 20–200 ppm CdCl_2_·2.5 H_2_O (Table 1) and incubated aerobically at 30°C for 7 days. Secondary enrichments were grown under the same conditions. These enrichments were then plated at a 10^−5^ dilution on 0.5 × Nutrient Agar amended with Cd (20–200 ppm). Isolated colonies were purified with multiple transfers and identified using colony PCR of the 16S rRNA gene with ProMega GoTaq Master Mix and primers 27Fm and 1492R (6), followed by Sanger sequencing at Eurofins Genomics (Louisville, KY, USA). Results from a BLASTn (2.16.1+) (7) web interface search of the nonredundant nucleotide database (nr/nt) were used to classify each sequence.

**TABLE 1:** Genome-assembly data and genetic determinants of cadmium-resistance

Isolate									
Genome characteristic	EM01	EM02	EM03	EM06	EM08	EM09	EM17	EM32	EM12
SRA accession #	SRS18577255	SRS18577256	SRS18577257	SRS18577258	SRS18577259	SRS18577260	SRS18577262	SRS18577263	SRS18577261
Genus-level taxonomy	*Methylobacterium*	*Methylobacterium*	*Methylobacterium*	*Methylobacterium*	*Methylobacterium*	*Methylobacterium*	*Methylobacterium*	*Methylobacterium*	*Methylorubrum*
Sample latitutude	36.9871	36.9871	36.9871	36.9871	36.9871	36.9871	36.9871	36.9871	36.9871
Sample longitude	−94.8028	−94.8028	−94.8028	−94.8937	−94.8028	−94.8028	−94.8028	−94.8028	−94.8937
[Cd] in enrichment (ppm)	150	150	150	150	200	200	200	20	200
Assembler, *k*-mer used	SPAdes, 99	SPAdes, 99	SPAdes, 99	SPAdes, 97	SPAdes, 99	SPAdes, 99	SPAdes, 99	SPAdes, 97	SPAdes, 97
Total # of reads	2,867,980	2,717,565	2,861,174	1,248,534	2,232,758	1,248,480	2,614,436	3,888,477	2,233,578
Assembly size (nt)	5,859,422	5,857,155	5,859,060	6,582,411	5,933,092	5,929,852	5,929,105	7,427,470	5,645,895
Contigs	90	95	95	403	109	126	100	278	138
Completeness (%)	99.69	99.69	99.69	99.22	99.69	99.69	99.69	100	99.69
Contamination (%)	0	0	0	0.63	0	0	0	0.94	0
GC content (%)	69.85	69.84	69.85	69.69	69.73	69.73	69.74	70.46	69.98
*N*_50_	143,407	138,670	146,637	34,111	142,727	116,000	142,609	51,914	76,531
*L*_50_	14	13	13	55	16	18	13	46	24
Total # genes	5,624	5,624	5,726	6,688	5,725	5,838	5,708	6,884	5,266
Protein-coding genes	5,551	5,551	5,646	6,605	5,650	5,755	5,633	6,771	5,199
RNA genes	73	73	68	83	75	12	75	113	67
Cadmium export gene *czcB*	3	3	3	5	3	3	3	3	3
Cadmium export gene *czcA*	3	3	3	4	3	3	3	3	3
Cadmium export gene *czcC*	1	1	1	1	1	1	1	1	1
Cadmium export gene *czcD*	2	2	2	1	2	2	2	2	2
Cd^2+^/Zn^2+^-exporting ATPase	1	1	2	2	1	1	1	2	2
Cadmium resistance transporter	0	0	0	0	0	0	0	1	0
Phytochelatin synthase	1	1	1	1	1	1	1	0	0

Isolates of interest were grown in the enrichment conditions described above for 2 days, pelleted, and sent to The Sequencing Center (Ft. Collins, CO, USA). DNA extraction was completed using the Zymo Quick-DNA Fungal/Bacterial kit, and library preparation (Nextera XT kit) and Illumina sequencing (MiniSeq Reagent Kit High Output, 2 × 150 bp) was performed according to the manufacturer’s protocols.

Sequences were quality filtered using BBTools’ (v38.32; sourceforge.net/projects/bbmap/) bbduk script (ftr = 149 qtrim = r maxns = 1 trimq = 6 maq = 8 minlength = 51). Reads were assembled using SPAdes (v3.10.1) (8) with *k*-mer values ranging from 25 to 99 in steps of 2. Optimal assemblies (Table 1) were identified based on *N*_50_ values calculated using Quast (v5.0.1) (9). Afterward, contigs shorter than 1 kb were manually removed from the assembly. Human contamination (human genome v38) was assessed using BLAST+ (v2.7.1+) (7) and removed manually. Genomes were also assessed (Table 1) using CheckM (v1.0.13) (10). Annotations were performed using the DOE-JGI Microbial Genome Annotation Pipeline (MGAP v.4) (11) and Integrated Microbial Genomes (12) was used to further analyze genes related to Cd resistance (Table 1). Default parameters were used for all programs unless otherwise noted.

**Fig 1:**
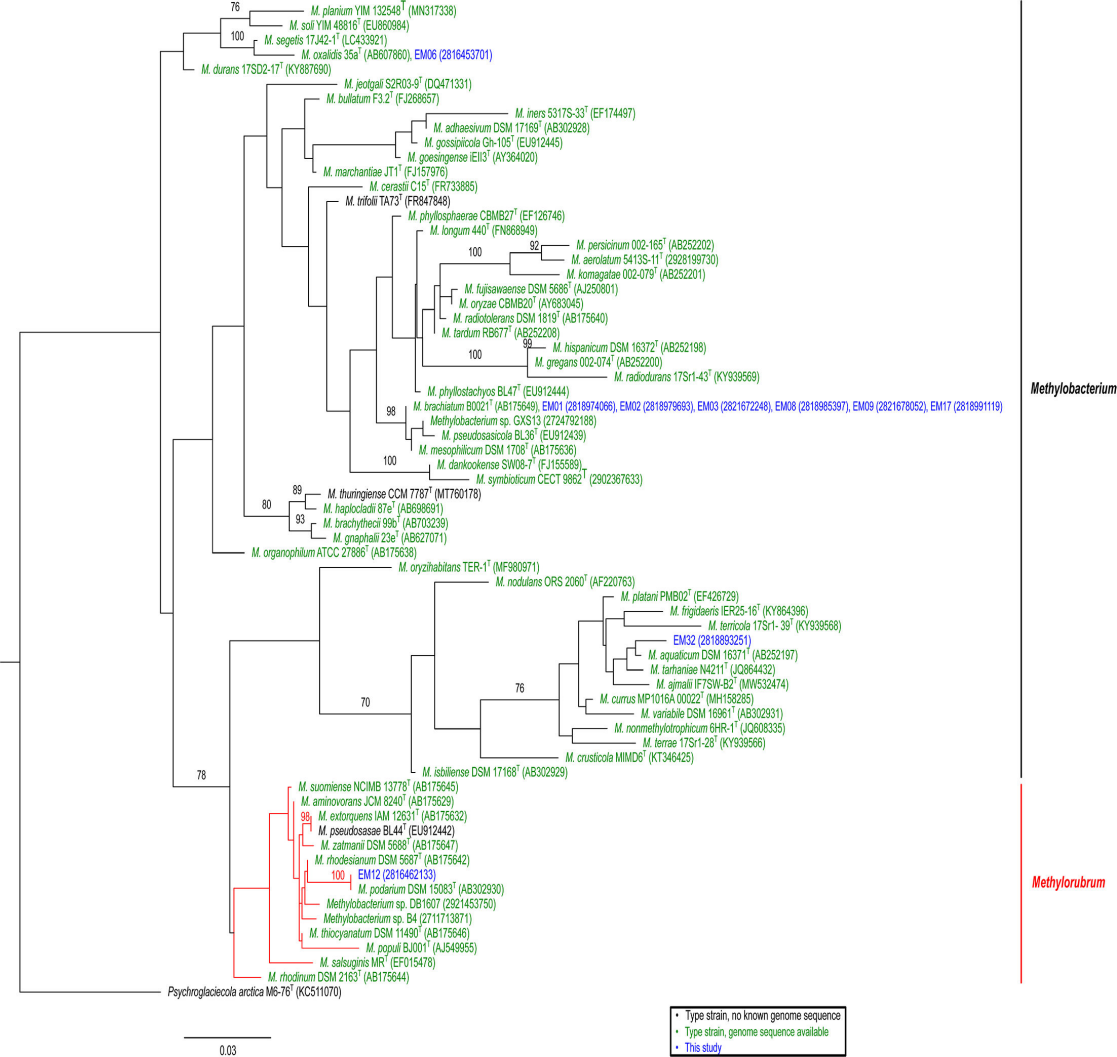
Maximum likelihood phylogeny of 16S rRNA genes. MUSCLE (14) within Mega11 (15) was used to align sequences. MEGA was also used to identify GTRGAMMA + I as the optimal substitution model. RAxML (13) was used to create a phylogeny with 1000 bootstrap replicates. Labels for nodes with less than 70% bootstrap support were removed. All validly named species of *Methylobacterium* and *Methylorubrum* were included. Sequenced genomes are shown in green text, unsequenced type strains are shown in black text and our strains are depicted in blue text. NCBI (alphanumeric) or IMG accession numbers (numeric) are listed in parentheses. When sequences were identical, one sequence was retained from the alignment for construction of the phylogeny, as suggested by RAxML documentation (13). Subsequently, names of sequences removed were manually added to the tree during annotation. The scale bar represents nucleotide substitutions per site.

A maximum likelihood phylogeny of 16S rRNA genes of *Methylobacterium* and *Methylorubrum* type strains (Fig. 1) created in RAxML (v8.2.11) (13) indicated these isolates are most closely related to type strains *Methylobacterium oxalidis* (EM06), *Methylobacterium brachiatum* (EM01, 02, 03, 08, 09, and 17), *Methylobacterium aquaticum* (EM32), and *Methylorubrum podarium* (EM12).

Several Cd-resistance proteins encoded in these genomes are similar to those employed by other genera, such as CzcCBA, Cd^2+^/Zn^2+^-exporting ATPases, and the CzcD cation diffusion facilitator family transporter (16) (Table 1). In addition, all genomes except EM12 and EM32 encode a phytochelatin synthase, a protein that has been shown to be expressed in the presence of Cd in other prokaryotes (17) and can result in increased tolerance to certain heavy metals and other stressful environments (18). Presence of these genes suggests that isolates may tolerate high Cd levels via a combination of efflux and sequestration.

## Data Availability

Illumina sequencing files have been deposited in the SRA under BioProject PRJNA1004332. Individual SRA accession numbers are listed in Table 1. Annotation files can be accessed at https://doi.org/10.5281/zenodo.14251494.
